# Assay of Desmopressin Acetate in Nasal Spray: Development of Validated Pre Column HPLC-Fluorescence Method

**DOI:** 10.15171/apb.2017.054

**Published:** 2017-09-25

**Authors:** Neeraj Upmanyu, Pawan Kumar Porwal

**Affiliations:** ^1^School of Pharmacy & Research, People's University, By-Pass Road, Bhanpur, Bhopal (M.P.)-462037, India.; ^2^Department of Pharmaceutical chemistry, SNJB’s SSDJ College of Pharmacy, Chandwad (Maharashtra)-423 101, India.; ^3^Department of Quality Assurance, ISF College of Pharmacy, Moga, Punjab-142001, India.

**Keywords:** Desmopressin acetate (DDAPV), Ortho-Phthaldehyde, Spectrofluorometric, HPLC-Fluorescence, Derivatization, Nasal spray

## Abstract

***Purpose:*** Desmopressin acetate (DDAPV), a synthetic analogue of vasopressin, has been recommended to be used in diabetes insipidus, mild forms of hemophilia and Von Willebrand disease. The DDAPV is available for adminstration via different routes viz. oral, parenteral and nasal, however its dose is very less in case of nasal sprays (20 µg) and parenteral route (4 µg) compared to oral route (0.1 to 0.3 mg in tablet). A sensitive and selective method is needed to be developed and validated for assay of low concentrations of DDAPV in its pharmaceutical dosage form i.e. nasal spray.

***Methods:*** Simple and specific HPLC-Fluorescecne method has been proposed for the quantitation of DDAPV at nanogram level in nasal formulations for the first time. DAPV, DDAPV EP impurity-B, chlorobutanol, benzalkonoium chloride were successfully derivatised with Ortho-Phthalaldehyde (OPA) and co-eluted on a C8 (50×2.1 mm, 3.5 µm particle size, 120Å) with mobile phase composed of 0.1% trifluroacetic acid, acetonitrile and Isopropyl alcohol in ratio of 70:25:5. The emission was measured at 450nm and flow rate was 0.8ml/min. The reaction was optimized in the terms of pH, stability of formed fluorophore and time consumed during the reaction.

***Results:*** The maximal fluorescence intensity was reached when the solutions were mixed for 3 min, and remained constant for at least 30 min at 20-25ºC. The calibration curve was found linear from 50 to 5000 ng/ml with weight of 1/X^2^. The limit of detection was 10ng/ml and precision was less than 2.0.

***Conclusion:*** The developed HPLC-fluorescence assay method was successfully applied for quantitation of DDAPV in nasal spray. HPLC-Fluorescence method was specific, sensitive, precise and accurate for determination of DDAPV. The method was able to quantify DDAPV at 50ng/ml with sufficient accuracy and precision. The validated HPLC-Fluorescence was successfully applied.

## Introduction

Desmopressin acetate (DDAPV), a synthetic analog of vasopressin, is used in the treatment of central diabetes insipidus,^1^ mild forms of hemophilia^2^ and Von Willebrand disease.^3^ The major side effect of DDAPV is completed dryness especially in minority.^4^ DDAPV nasal spray has been recommended for effective control over bleeding episodes or less heavy menstrual in women compared to conventional route of administration.^5^ DDAPV nasal spray has also been recommended for treatment of bladder dysfunction in patients with multiple sclerosis.^6^ DDAPV is available as a formulation via different routes, however its dose is very less in case of nasal sprays (20 µg *i.e.* 10 µg per 0.1ml) and parenteral route (4 µg) compared to oral route (0.1 to 0.3 mg tablets).

Though several analytical methods have been reported in literature for determination of DDAPV simple, accurate and robust determination of DDAPV is still matter of difficulty.^7^ DDAPV is a small but highly basic peptide and unstable at high pH. Major analytical problem with DDAPV is its low UV absorption which create obstruction in development of simple HPLC-UV method. Aside highly sensitive method is required for quantitation of DDAPV in nasal spray. Limit of detection for HPLC-UV methods were 10µg/ml^8^ and 25µg/ml,^9^ which make HPLC-UV methods unsuitable for quantitation of DDAPV in nasal spray. Second analytical problem, which eliminate suitability of spectral method for assay of DDAPV in nasal spray, is presence of Preservatives *viz.* chlorobutanol^10^ and benzalkonium chloride (BKC)^11^ in nasal spray formulation. Liquid chromatography coupled with tandem mass spectrometry methods have been reported to quantify at DDAPV down to picogram concentration level.^7,12-15^ Though LC-MS/MS methods provide sufficient accuracy and sensitivity and useful for analysis of DDAPV in nasal spray and in biological fluid, instrument cost play a crucial role for usage of this analytical technique more frequently. High basicity of DDPAV due to prolin substituted nitrogen group provides an opportunity to convert DDAPV into fluorophore with some fluorescence derivatising agent *viz.* Ortho-Phthalaldehyde (OPA) or chloroformates.^16^

Given this as background, it was thought worthy to develop fast, economical, sensitive, accurate and precise analytical method for quantitation of DDAPV in nasal spray in the presence of common preservative. The UV-Spectroscopic, spectrofluorometric, HPLC-UV and HPLC-Fluorescence methods are needed to be optimized for assay of DDAPV in nasal spray in the presence of common preservative.

## Materials and Methods

### Reagents and chemicals

Qualified standard of DDAPV was a gift sample from Ranbaxy Research laboratory (Gurgaon, India). DDAPV EP Impurity B was purchase from Clearsynth^®^ Asia research centre (Mumbai, India). OPA reagent, BKC and chlorobutanol and other reagent were purchased from sigma Aldrich (Bangalore, India). Analytical/HPLC grade chemicals and solvents were obtained from Ranbaxy Fine Chemicals Limited (Delhi, India). Deionized and splash distilled water (Conductivity: 16.5mΩ) was prepared in house and filtered through 0.22µm filter.

### Instrumental conditions

The Chromatograph consisted of a JASCO 2000 series HPLC System equipped with 2089 Quaternary Pump, UV-2075 UV-Detector and FP- 2020 Fluorescence detector, AS2059 Autosampler, and LC-Net II ADC Controller. The Chromatographic data were evaluated by ChromPass™ Software. A Shimadzu RF- 5301PC spectrofluorometer (Kyoto, Japan) was utilized for Fluorescence detection, whereas a Shimadzu 1800 spectrophotometer (Kyoto, Japan) was used for UV-spectroscopy measurements.

Each analyte (DDAPV and preservatives) was scanned from 400-200nm to get UV-spectrum and UV-absorptivity was calculated at wavelength maxima. The DDAPV was derivatised with Ortho-Phyldehyde reagent solution and in 1:1 ratio. The excitation wavelength was 340nm whereas, emission was recorded at 445 nm for DDAPV-OPA complex. The recoveries of DDAPV-OPA complex was optimized in terms of different variables viz. buffer concentration, OPA concentration and reaction time.

HPLC-UV method involve elution of DDAPV on Kromasil C8 (150×4.6mm, 5 µm particle size) as stationary phase and mobile phase was consisting of 0.1% tri fluoro acetic acid (TFA) with Acetonitrile (ACN) in the ratio of 75: 25. The flow rate was 1.0ml/min and elution was monitored at 220nm using UV-absorbance detector. For HPLC-fluorescence method Optimum separation conditions were obtained with a SunFire C_8_ (50 × 2.1 mm i.d. with 3.5 μm particles, 100Å pore size) column with mobile phase consisting of 0.1 % TFA: Acetonitrile (ACN): Isopropyl Alcohol (IPA) in the ratio of 70:25:5 with column oven temperature maintained at 30°C and elution monitored by a emission wavelength detection at 455 nm. The JASCO AU-2059 autosampler was used for automatic derivatization of DDAPV (50µL) by adding 500 µL of OPA reagent to an autosampler vial. The reaction was allowed to happen for 3min for completion of derivatization prior to injection. All measurements were performed with an injection volume of 10 μl at 25ºC autosampler temperature.

### Preparation of Solutions

#### Preparation of OPA solution

About 600 mg of OPA reagent was dissolved in 5 ml methanol and volume made to 50 ml with 100 mM borate buffer. Further, 5mL of OPA reagent was mixed with 15 mL of 2-Mercaptoethanol.

#### Preparation of DDAPV standard and resolution solution

DDAPV stock solution was prepared by taking appropriate quantity of analyte and dissolve using distilled water in a 10mL volumetric flask to get concentration of 1000µg/ml. Serial dilutions were made to get 10µg/ml concentration of DDAPV. The standard solutions for chlorobutanol, DDAPV EP impurity-B and BKC were prepared in appropriate solvent/s.

The OPA reagent was added to standard solution of in the ratio of 1:10 for analyte standard solution and OPA reagent respectively. Resolution solution (for Spectrofluoremetric and HPLC-fluorescence experiments) containing all analytes *i.e.* DDAPV, chlorobutanol, DDAPV EP impurity-B and BKC, were prepared in distilled water from their respective stock solution to get a final concentration of 500, 100000, 50 and 1000 ng/ml, respectively. Whereas the resolution solution, for UV-spectroscopic and HPLC-UV experiments, was prepared for all analytes *i.e.* DDAPV, chlorobutanol and BKC, were prepared in distilled water from their respective stock solution to get a final concentration of 10, 500, and 10 µg/ml, respectively.

#### System suitability

The HPLC-Fluorescence and HPLC–UV methods were optimized in the terms of system suitability parameters^17^*viz.* %RSD for peak area (n=6), %RSD for peak retention time (n=6), capacity factor (k’= t_r_-t_m_/t_m_), no. of theoretical plates per meter (n= 5.54(t_r_/W_0.5_)^2^), peak asymmetry factor (P_As10%_ = B/A) and resolution (Rs = 2(t_r2_-t_r1_)/W_1_+W_2_) between any two closely eluting peaks.

#### Calibration curve

Calibration curve for DDAPV was plotted from 50 to 5000 ng/ml. The linearity graph was plotted between mean peak area (n=3) and concentration and statistical treatment was performed for calibration curve. The standard curve was prepared for 50, 100, 300, 500, 1000, 2000, 5000 ng/ml of DDAPV. The *goodness of fit* was observed for linearity curve and models for weighing (1/X and 1/X^2^) were employed to access relative error at lower concentration.

#### Specificity and Sensitivity

Specificity was determined for DDAPV in the term of non-interference at the retention time of DDAPV due to blank, placebo, and preservative/s present in placebo. The DDAPV EP impurity-B was spiked to DDAPV standard solution at 50 ng/ml concentration level. Similarly interference was observed at the retention time of chlorobutanol and BKC. Sensitivity was observed in terms of Limit of Detection (LOD) and Limit of Quantitation (LOQ) as per IUPAC method. Serial dilutions were made and concentration was back calculated using *corrected* calibration curve line equation. The %RSD value was considered as determination factor for sensitivity.

#### Accuracy and precision

Repeatability, reproducibility and the accuracy were calculated from data obtained during a 6-day validation. Three concentrations were chosen from the high medium and low range of the standard curve (100, 500, 2000 ng/ml) for DDAPV. Accuracy exercised using recovery studies and the results were expressed as the mean relative error (%RE). Whereas precision value (%CV) less than or equal to 2% for analyte were acceptable.

A comparative profile was generated for UV-spectroscopic, spectrofluorometric, HPLC-UV and HPLC-fluorescence methods for determination of DDAPV, method complexity, Specificity, sensitivity and calibration range.

#### Application of the analytical method

DDAPV was assayed in bulk and finished pharmaceutical product (FPP) *i.e.* nasal spray. The label claim for FPP was 10 µg/0.1ml of DDAPV. Two nasal spray puffs were collected in round head of 5ml volumetric flask for each measurement. The collected sample was washed with distilled water and volume was made up to the mark with same. The sample solution was further diluted and mixed with OPA reagent to get a test concentration 500ng/ml of DDAPV. The samples were prepared in duplicate and filtered using 0.45 µm dispo nylone syringe filters. The assay was calculated using following formula.

$$\%\;Assay=\;\frac{Average\;peak\;area\;of\;sample}{Average\;peak\;area\;of\;standard}\times \frac{weight\;of\;standard}{standard\;dilution\;}\times \frac{sample\;dilution\;}{weight\;of\;sample}\times \frac{standard\;potency}{lable\;calim}\;\times factor$$

## Results and Discussion

### Development of spectroflurometric method 

**Figure 1:**
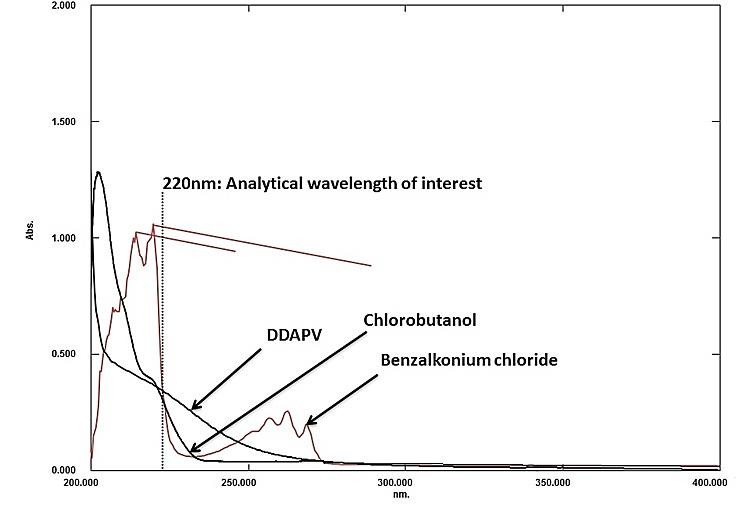
Overlain UV spectra of DDAPV, chlorobutanol and BKC

**Figure 2:**
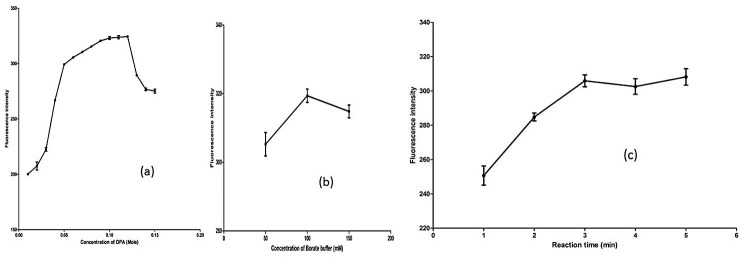
Effect of (a) OPA Concentration, (b) Borate buffer concentration and (c) reaction time on fluorescence intensity

As discussed earlier, the major analytical problems associated with sensitive determination of DDAPV is its low specific UV absorbance, possible interference due to probable formulation excipient present in nasal spray. The overlain UV-spectra of DAPVV with chlorobuatnol and benzalkonium chloride (both preservative) were shown in Figure 1. As shown in figure the UV-absorption Spectrum of DDAPV was completely overlapped by UV-Spectrum of BKC. The UV- spectrum of Chlorobutanol had shown a strong interference over the UV spectrum of DDAPV. The instrument’s analytical response for DDAPV was not justifying its use for determination of analyte in nasal spray. Therefore, to get a sensitive and specific analytical method for assay of DDAPV in nasal spray, fluorophore was added to DDAPV and excipients using Ortho-Phthalaldehyde (OPA) as derivatising agent because of its unique selectivity toward primary amine. OPA is preferred over other fluorescence derivatising agent for protein and peptide. The alkaline media was generated using borate buffer. Potassium borate was preferred over sodium borate due to its low back ground noise. The experimental results indicated that the maximum and constant fluorescence intensity of derivative has been observed, when OPA concentration was in the range 0.06-0.12M, hence 0.09M of OPA was taken as optimal concentration. The excitation wavelength was 340nm whereas, emission was recorded at 455nm. Optimum fluorescence intensity was observed at 0.09M OPA (Figure 2a), 100mM borate buffer concentration (Figure 2b) and optimum reaction time was 3.0 min (Figure 2c). The formed OPA - DDAPV complex was found stable at pH value of 9.0, 10.0 and 12.0 and DDAPV complex was found stable and minimal decrease in fluorescence intensity was observed for first one hour.

**Figure 3:**
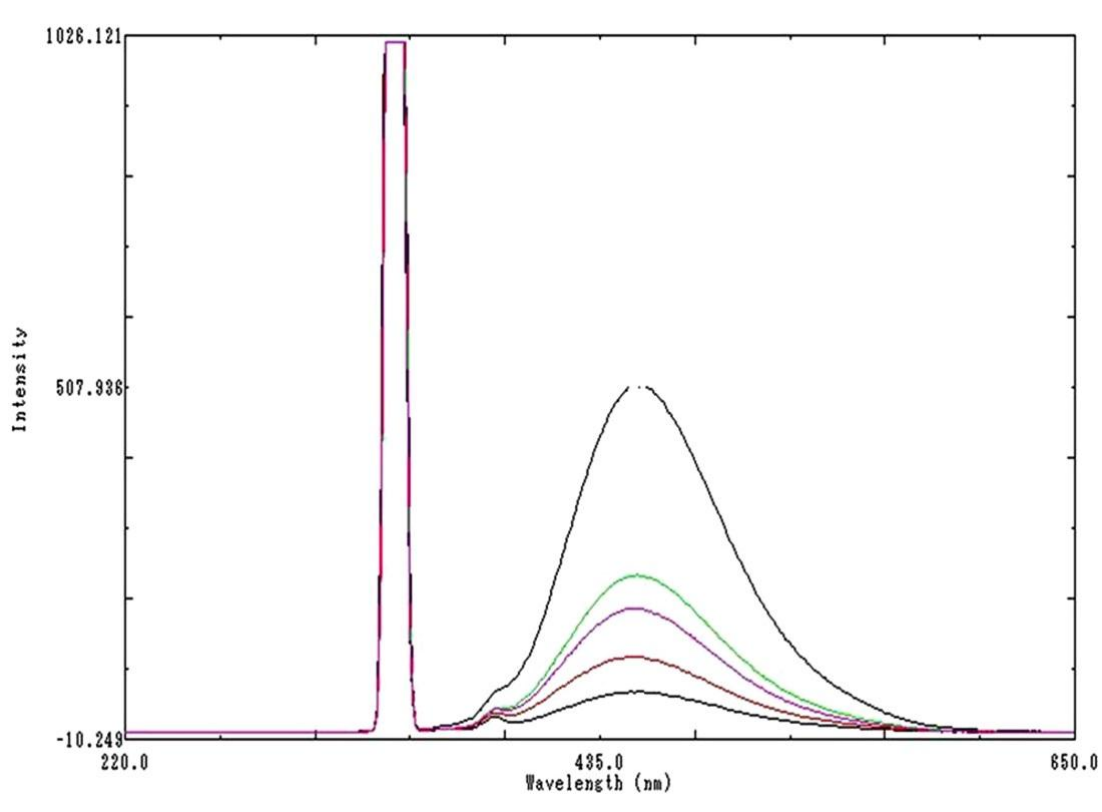
Overlain calibration spectra of DDAPV at 445nm emission wavelength

The studies were performed in the presence of chlorobuatnol and BKC. Though the mechanism was not clear, improved recoveries for OPA-DDAPV complex were obtained in the presence of BKC. An overlain emission spectrum showing calibration concentration in the range from 50 to 1000 ng/ml was given in Figure 3.

### Development of HPLC-Fluorescence method 

**Figure 4:**
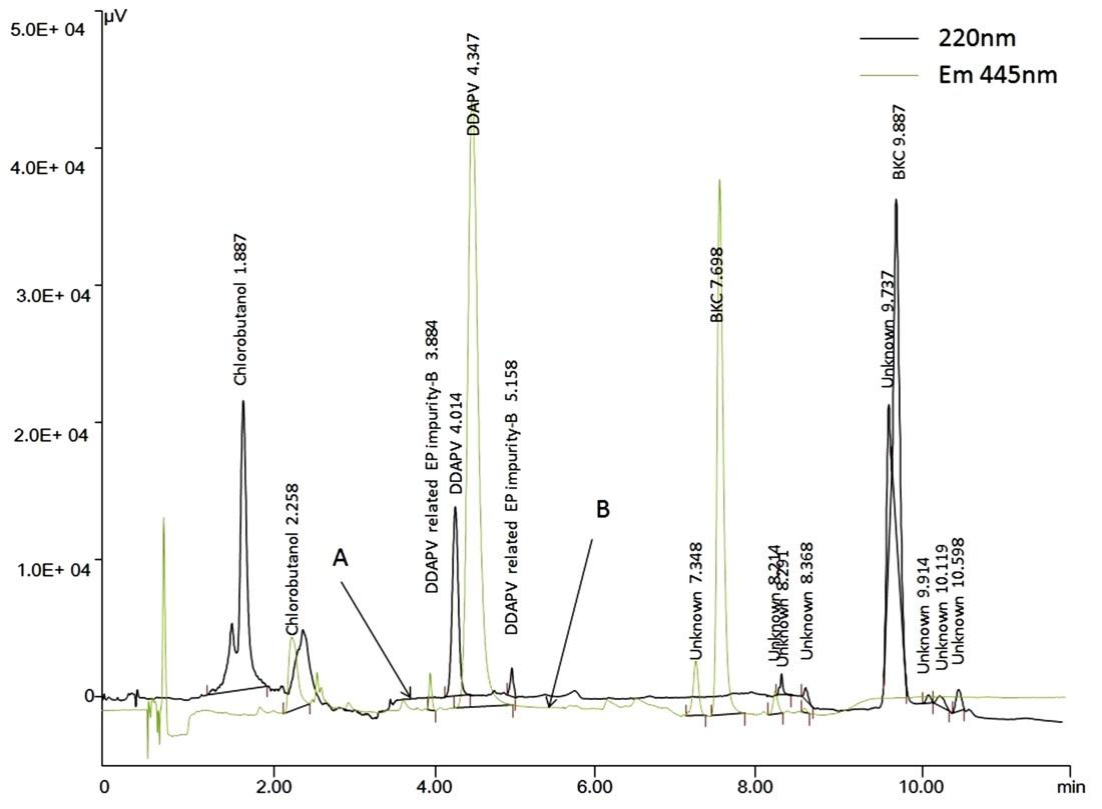
Overlain chromatogram of elution pattern for DDAPV, chlorobutanol, and BKC in (a) HPLC-UV and (b) HPLC-Fluorescence optimized methods

Initial HPLC conditions for co-elution of DDAPV, chlorobutanol and BKC, were adopted from the HPLC methods those were reported in literature. In the method reported in literature,^9^ the DDAPV and chlorobutanol was eluted within 10min, but it had taken more than 30min for elution of BKC, when employed in the laboratory conditions. When, the HPLC conditions employed as mentioned in literature,^18^ the elution of Chlorobutanol, DDAPV and BKC, the Chlorobutanol was eluted in column void and DDAPV was eluted at 1.86 min whereas, BKC was eluted within 10min. Therefore, in the next trial 0.1 %TFA with ACN in the ratio of 65:35 was used as mobile phase on a C18 stationary phase. The run time was more than 15min, therefore, ACN was increased in organic phase component of mobile phase was increase from 30% to 40% to reduce the elution time of BKC as shown in Figure 4. The UV absorptivity of DDAPV was less than 20 thus sensitivity (LOQ) of the developed HPLC-UV method was about 10 µg/ml.

The UV-detection response of HPLC-UV method for DDAPV was not meeting the goal of analytical method (*i.e.* assay of DDAPV in nasal spray). Hence an *online* precolumn fluorescence derivatization of the DDAPV was exercised using OPA as fluorescence derivatizing agent. The OPA-reagent was mixed with 2-mercaptoethanol in the ratio of 1:2, 1:1 and 1:3, respectively and optimum fluorescence intensity was obtain when the ratio for OPA reagent and 2-mercaptoethanol was 1:1. The OPA reagent solution was automatically mixed with sample (containing DDAPV) in equal volume (e.g. 1000 µL of OPA and 100 µL of DDAPV) in a vial and reaction was allowed to ensue for 3.0 min followed by injection of resulting solution to chromatograph using autosampler. The stability of DDAPV –OPA complex was more than one hour (as prepared in earlier section); therefore the need of post column derivatization was eliminated.

The elution of DDAPV-OPA complex was optimized on C8 with wide pore size (≈120 Å) using 0.1% TFA and acetonitrile in the ratio of 60: 40, respectively. The retention time for Chlorobutanol, DDAPV and BKC were 2.55, 8.54 and 11.42 min, respectively. Though each peak had passed System suitability parameter, the resolution between an unknown peak and BKC was less than 2.0, and peak was showing a small but notable shoulder in the peak front. In the next trial, second organic modifier (*i. e.,* Isopropyl alcohol; IPA) was added to the mobile phase. With addition of IPA (~ 5%) as organic modifier, it has been observed that previous BKC peak was sub-dived in three peaks (RRT 0.99 and 1.11 with respect to BKC peak). Later, the unknown peaks of RRT 0.99 and 1.11 were identified as BKC homologous impurities. Therefore final HPLC-Fluorescence method was consisting of simultaneous elution Chlorobutanol, DDAPV and BKC on a Sun Fire C8 (50×2.1 mm, 3.5 µm particle size) column using 0.1%TFA, acetonitrile and IPA in the ratio of 70:25:5, respectively as mobile phase. The elution was monitored at 445 nm and flow rate was 0.8 ml/min.

The developed HPLC-Fluorescence was optimized in the terms of system suitability parameters. The results for system suitability parameters viz. %RSD for peak area (n=6), %RSD for peak retention time (n=6), capacity factor, no. of theoretical plates, peak asymmetry factor and resolution between any two closely eluting peaks were summarized in Table 1.

**Table 1:** System suitability and other peak parameters for DDAPV for HPLC-Fluorescence and HPLC-UV method (n=6)

**System suitability Parameter**	**HPLC-Fluorescence**	**HPLC-UV**
**Mean peak area**	455489 (500ng/ml)	245285 (10 µg/ml)
**Retention time**	4.34	4.01
**% RSD for peak area**	1.49	1.19
**%RSD for Retention time**	0.64	0.51
**Capacity factor**	4.88	4.34
**Peak asymmetry factor**	1.44	1.21
**Number of theoretical plates**	4,708	2998
**Resolution between DDAPV and its related impurity**	2.68	4.14
**Resolution between BKC and its homologous impurity**	2.11	1.69

### Calibration curve 

Calibration curve for DDAPV was plotted from 50 to 5000 ng/ml. The models for weighing were employed to observe *goodness of fit* and relative error was calculated at lower concentration. Table 2 had enlisted various linearity parameters of unweight, 1/X and 1/X^2^ weighed calibration curve.

As shown in table, the values for correlation coefficient were more than 0.997 for all calibration curves but mean relative error (n=3) was highest for unweight calibration curve and lowest for 1/X^2^. Therefore, weighted calibration curve (1/X^2^) was employed for linearity validation.

**Table 2:** Comparison of Weighted and Unweighted calibration curves for DDAPV

**Cali. Curve** **( ng/mL)**	**Unweighted linearity curve**					**1/X** ^ 2 ^ ** weighted linearity curve**				**1/X** ^ 2 ^ ** weighted linearity curve**			
	**m**	**c**	**Sy.x**	**r** ^ 2 ^	**%MRE**_LOQ_	**m**	**c**	**SE**_c_	**%MRE**_LOQ_	**m**	**c**	**SE**_c_	**%MRE**_LOQ_
50-5000	25.28	-1.91 × 10^-2^	0.225	0.997	25.656	1.998	6.25× 10^-2^	1.11× 10^-2^	12.25	4.995	2.21 × 10^-3^	2.7 × 10^-3^	5.251

m and c are slope and y- intercept, respectively, for line equation of y=mx+c. SE_c_ is standard error of Y-intercept.And **%MRE**_LOQ_is %Mean relative error at LOQ level

### Specificity and sensitivity 

**Figure 5:**
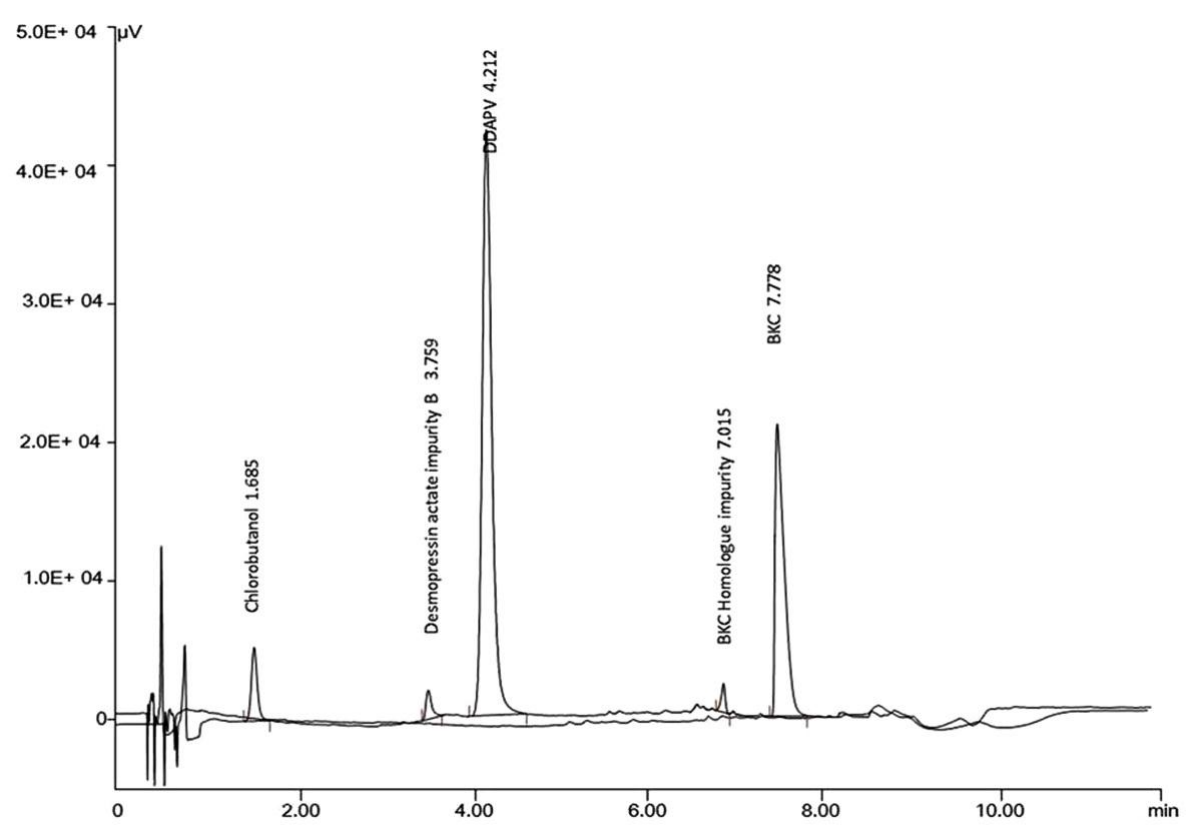
Specificity chromatogram for DDAPV showing noninterference at retention time overlain with blank

The HPLC-fluorescence chromatograms were recorded for DDAPV alone and with DDAPV related EP-impurity-B. The baseline was noisier for HPLC-fluorescence chromatograms compared to HPLC-UV Chromatogram but no interference were recorded at the retention time of DDAPV and its related impurity. The resolution between DDAPV and its impurity was more than 2. The resolution between BKC and its homologous impurities was also more than 2. The resolution between any two closely eluting peak was more than 2 and peak asymmetry factor of the peak for DDAPV and its impurity was always in the range 1.13–1.47, which indicate that the developed HPLC-fluorescence was specific for co-elution of DDAPV, its impurity, chlorobutanol, BKC and BKC homologous impurity as shown in Figure 5.

**Figure 6:**
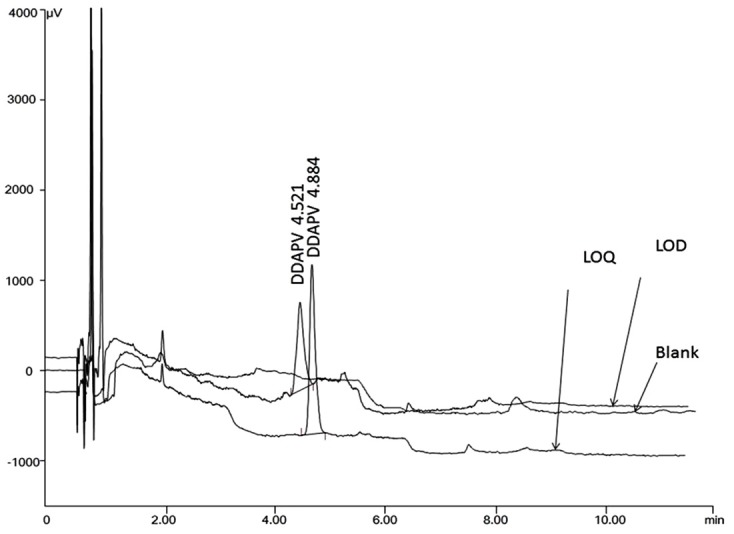
Overlain chromatogram of limit of detection (5ng/ml) and limit of quantitation (15ng/ml) for DDAPV

Sensitivity of the HPLC-fluorescence method was determined using IUPAC method. The sample was serially diluted and DDAPV was quantified using 1/X^2^ weighed calibration curve and %RSD was calculated at each concentration level. The optimized HPLC method was sufficient enough to detect (LOD) and quantify (LOQ) at 5 and 15 ng/ml concentration level, respectively with acceptable value of precision as shown in Figure 6. The %RSD value for LOD was 13.16 and 5.84, respectively. DDAPV- OPA complex have shown more sensitivity in the terms of LOD and LOQ for Spectrofluoremetry compared to HPLC –fluorescence method. Even more sensitive HPLC-fluorescence could be claimed but retention time of DDAPV varied with higher coefficient of variance at lower LOD and LOQ values for later, therefore, sensitivity values were kept higher.

### Precision and accuracy

Precision and accuracy studies were performed at low, medium and high level of calibration curve, therefore 100, 500 and 2000 ng/ml. The %RSD value for repeatability and reproducibility were 1.11 and 1.86, respectively. Percent recovery values of DDAPV for developed HPLC-fluorescence were in the range of 96.5-102.5% with mean value (± SD) of 101.1 (± 2.23).

### Application of method

**Figure 7:**
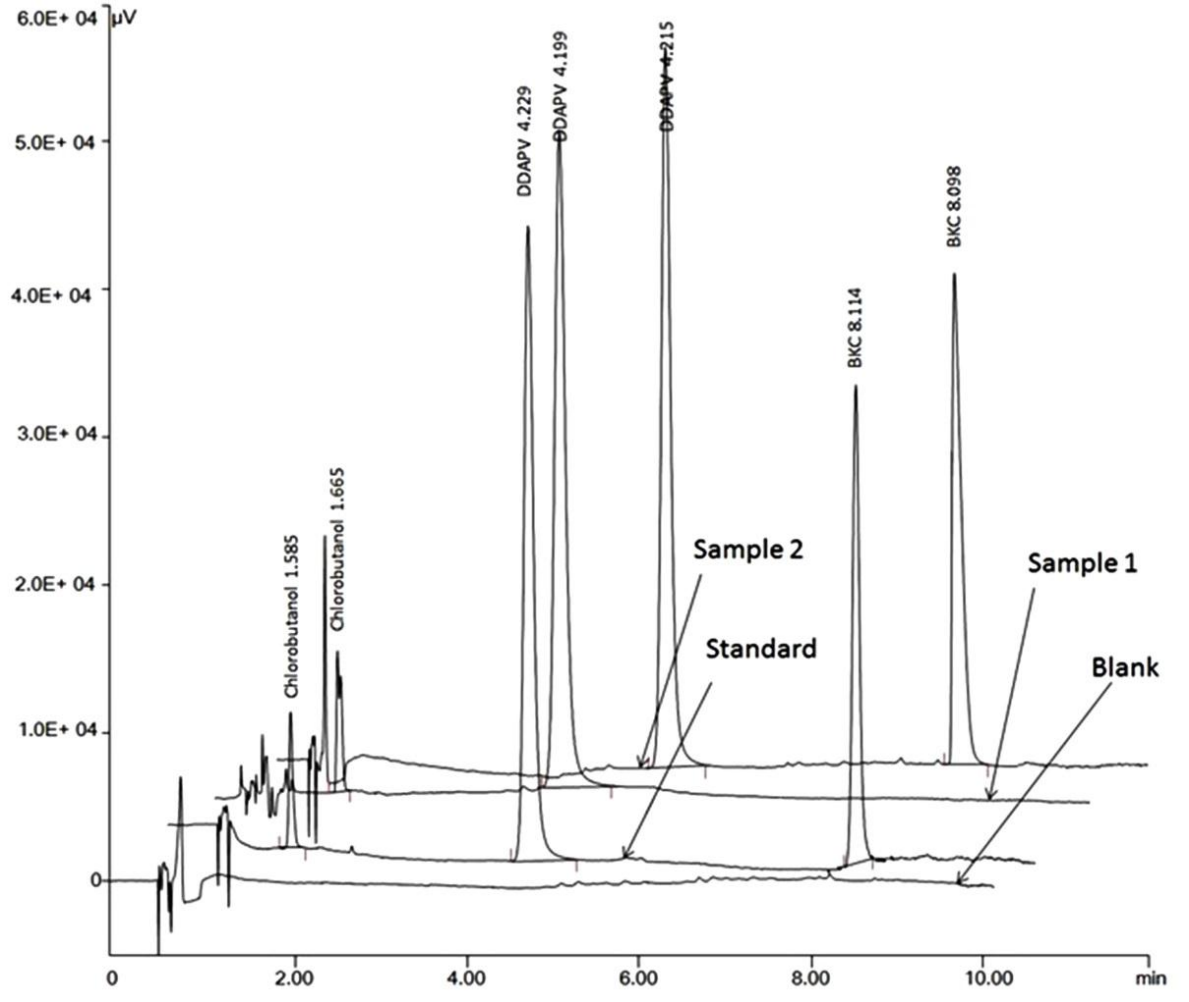
Overlain representative chromatograms for DDAPV in standard and marketed preparation containing chlorbutanol as preservative and BKC as preservative

The optimized HPLC method was applied for determination of DDAPV in bulk and in marketed dosage form. The % biases for DDAPV were in the range of -1.5 to 2.0 and mean assay value for DDAPV was 97.6 and 98.5 for marketed formulation-1 and marketed formulation-2. The results are given in Table 3 and an overlain chromatogram for standard solution and marketed formulations was given in Figure 7.

The UV-spectroscopic, spectrofluorometric, HPLC-UV and HPLC-fluorescence methods were compared for determination of DDAPV, method complexity, Specificity, sensitivity and calibration range. The results were depicted in Table 4.

**Table 3:** Assay results of DDAPV in bulk and marketed formulation using HPLC –Fluorescence method

**Bulk**			**Formulation**		
**Conc.**	**% Assay Value**	**Mean**	**Formulation**	**% Assay Value**	**Mean**
**100 ng/mL**	101.15	100.41±0.64	Formulation-1	99.1	97.8±1.3
**500 ng/mL**	100.5			96.5	
			Formulation-2	101.1	99.8±1.3
**2000 ng/mL**	99.58			98.5	

**Table 4:** Comparative profiles of various analytical methods for determination of DDAPV

**Parameter**	**UV-Spectroscopic**	**Spectroflurometric**	**HPLC-UV**	**HPLC-Fluorescence**
Test concentration*	**100 µg/ml**	**300ng/ml**	**10 µg/ml**	**500 ng/ml**
Specificity	+	+	++	+++
Sensitivity	+	+++	+	++
Calibration range	+	+++	++	+++
Derivative preparation	--	++	--	+++
Overall complexity	+	++	++	+++
Cost	+++	++	++	+++
Suitability of method for assay of DDAPV in nasal spray	+	+	++	+++

Where -- stand for not applicable, + for poor, ++ for medium and +++ stand for excellent method

## Conclusion

Four analytical methods were developed for determination of DDAPV and compared for its suitability for assay of analyte at lower concentration *i.e.* nasal spray. UV-Spectroscopic method was simple and economical but non-specific and least sensitive. The DDAPV-OPA Spectrofluorometric method was sensitive but not specific especially for the formulation containing BKC as preservative. Whereas the HPLC-UV method was found to be specific but that was not sensitive. The HPLC-UV method could be used assay of DDAPV in formulation, containing DDAPV in higher amount (i.e. tablets). HPLC-Fluorescence method was specific, sensitive, precise and accurate for determination of DDAPV. The method was able to quantify DDAPV at 50 ng/ml with sufficient accuracy and precision.

## Acknowledgments

The authors are highly thankful to sun pharmaceutical for providing gift sample of DDAPV and Clearsynth Asia (Mumbai, India) for providing DDAPV EP-impurity B at reduced rate. We are also thankful to principal and management of SSDJ College of Pharmacy for providing fund and research facility to carry out this work.

## Ethical Issues

Not applicable.

## Conflict of Interest

The authors declare no conflict of interests.
